# O Sistema Adenosina como Alvo na Busca de uma Nova Classe de Anti-Hipertensivos

**DOI:** 10.36660/abc.20240129

**Published:** 2024-04-19

**Authors:** Roberto Jorge da Silva Franco

**Affiliations:** 1 Universidade Estadual Paulista Júlio de Mesquita Filho Campus de Botucatu Faculdade de Medicina Botucatu SP Brasil Universidade Estadual Paulista Júlio de Mesquita Filho Campus de Botucatu Faculdade de Medicina, Botucatu, SP – Brasil

**Keywords:** Doenças Cardiovasculares/complicações, Mortalidade, Anti-Hipertensivos, Hipertensão, Adenosina

As complicações cardiovasculares (CV) são um dos principais fatores de mortalidade precoce no atual cenário mundial e tornaram-se grande desafio na busca do controle de sua ascensão tanto nos países em desenvolvimento quanto nos desenvolvidos. Tornou-se assim de imensa importância procurar diferentes possibilidades terapêuticas e tratamentos para o crescente fardo das doenças CVs. Os receptores de adenosina (AR) podem ser uma ferramenta inovadora de escolha na compreensão do mecanismo de sinalização que pode levar às complicações CVs.

A mediação da adenosina envolve a ativação de uma família de quatro AR acoplados à proteína G: A1, A2A, A2B e A3. O A3AR é o único subtipo de adenosina a ser super-expresso em células inflamatórias e cancerígenas, tornando-se assim um alvo potencial para terapia. A3AR apresentou uma natureza dupla sob diferentes condições fisiopatológicas e pode ser protetor/prejudicial em condições isquêmicas, pró/anti-inflamatório e pró/antitumoral dependendo nos sistemas investigados. Até recentemente, o maior e o desafio mais intrigante foi entender se, e em quais casos, agonistas A3 seletivos ou antagonistas seriam a melhor escolha.^1^

O nucleosídeo purina adenosina foi identificado como um importante regulador local da função tecidual, particularmente quando o fornecimento de energia celular não consegue atender o aumento das concentrações de adenosina sob condições metabólicas desfavoráveis. A hipóxia tecidual, por exemplo, leva a uma maior quebra de ATP e a um aumento da demanda de geração de adenosina. Esses receptores diferem em 1) sua afinidade pela adenosina, 2) o tipo de proteínas G a ser recrutado e, finalmente, 3) as vias de sinalização a jusante ativadas nas células-alvo. A1 e A3ARs inibem a regulação da atividade da adenil ciclase (AC), enquanto a ativação dos subtipos A2A e A2BAR estimula AC, ao que leva a aumentos nos níveis de AMP cíclico.^1^

Os ARs estão amplamente distribuídos por todo o corpo e o fato de estarem presentes basicamente em todas as células principalmente nos vasos, pulmões, rins, coração e partes do cérebro que torna um alvo interessante para intervenção farmacológica em muitas condições fisiopatológicas ligadas ao aumento níveis de adenosina. Há evidências que os A3ARs melhoram a capacidade antioxidante celular, assim contribuindo para a vasoproteção e redução da cardiopatia morte de miócitos e apoiando fortemente uma resposta cardioprotetora dependente de A3AR.^1^

Nesse número dos Arquivos Brasileiros de Cardiologia^2^ foram avaliados novos compostos N-acilidrazona que atuam sobre o sistema adenosina, compostos contendo selênio. Em particular, foi selecionado o LASSBio-2062 com ação agonista, vasodilatador potente através da ativação AR A3 e dos canais K.

O LASSBio-2062 (30 μmol/kg) reduziu a pressão arterial média em ratos SHR de 124,6 ± 8,6 para 72,0 ± 12,3 mmHg (p < 0,05). A ativação do receptor de adenosina subtipo A3 e dos canais de potássio parece estar envolvida no efeito anti-hipertensivo do LASSBio-2062. Em conclusão o novo agonista do receptor de adenosina e ativador dos canais de potássio é um potencial agente terapêutico para o tratamento da hipertensão arterial sistêmica.

Entre as diversas funções biológicas promovidas pelo selênio, tem sido descrita a ação antioxidante.^3^ A ativação dos AR A3 influencia a atividade dos canais de K+, especialmente dos canais de potássio sensíveis a ATP, podendo induzir sua abertura,^4^ o que resulta em hiperpolarização e consequente bloqueio dos canais de Ca^2+^. A redução do influxo de cálcio leva à menor concentração intracelular de cálcio e resulta em vasodilatação.^5^

A ocorrência de bradicardia após administração intravenosa de LASSBio-2062 pode ser benéfica devido à ausência das características de taquicardia reflexa de muitos medicamentos vasodilatadores que, além disso, causam retenção de volume necessitando associação de betabloqueador e diurético.

Este estudo sugere que o sistema adenosina pode ser uma nova classe de anti-hipertensivos no arsenal de medicamentos no tratamento da hipertensão arterial. Um dos predicados para o benefício dos anti-hipertensivos não pode ser atribuído somente à redução da pressão arterial. Devem ter também efeitos pleiotrópicos de proteção endotelial e de órgãos alvo presente no LASSBio-2062^2^ pelas suas características antioxidantes, anti-inflamatórias e atenuação da aterosclerose.

A maioria da ação dos anti-hipertensivos estão associado a bloqueio ou antagonismo dos sistemas hipertensores. Tentativas de vasodilatação foram feitas com agonistas da angiotensina 1-7^6^ possivelmente alamandina compostos que se contrapõem ao vasoconstritor angiotensina II. Ação agonista do receptor de adenosina para promover vasodilatação e reduzir a pressão arterial em humanos hipertensos é um desafio do LASSBio-2062 para consolidar-se como nova classe de anti-hipertensivos.
