# Supplementary material for: O Sistema Adenosina como Alvo na Busca de uma Nova Classe de Anti-Hipertensivos
Source: Arq Bras Cardiol. 2024 Apr 19;121(2):e20240129. [Article in Portuguese] doi: 10.36660/abc.20240129 (PMC11081398; doi:10.36660/abc.20240129)
Supplement: Translation

# Translation: The Adenosine System as a Target in the Search for a New Class of Antihypertensives

**Authors:** Roberto Jorge da Silva Franco

**Affiliations:** 1 Botucatu SP Brazil Universidade Estadual Paulista Júlio de Mesquita Filho Campus de Botucatu Faculdade de Medicina, Botucatu, SP – Brazil

**Keywords:** Cardiovascular Diseases/complications, Mortality, Antihypertensive Agents, Hypertension, Adenosine

Cardiovascular (CV) complications are one of the main factors of early mortality in the current global scenario and have become a major challenge in the search to control their rise in both developing and developed countries. It has therefore become immensely important to look for different therapeutic possibilities and treatments for the growing burden of CV diseases. Adenosine receptors (AR) may be an innovative tool of choice in understanding the signaling mechanism that can lead to CV complications.

Adenosine mediation involves the activation of a family of four G protein-coupled AR: A1, A2A, A2B, and A3. A3AR is the only adenosine subtype to be overexpressed in inflammatory and cancer cells, thus making it a potential target for therapy. A3AR showed a dual nature under different pathophysiological conditions and could be protective/harmful in ischemic conditions, pro/anti-inflammatory, and pro/antitumor depending on the systems investigated. Until recently, the biggest and most intriguing challenge was understanding whether, and in which cases, selective A3 agonists or antagonists would be the best choice.^1^

The purine nucleoside adenosine has been identified as an important local regulator of tissue function, particularly when cellular energy supply cannot meet increased adenosine concentrations under unfavorable metabolic conditions. Tissue hypoxia, for example, leads to greater ATP breakdown and increased demand for adenosine generation. These receptors differ in 1) their affinity for adenosine, 2) the type of G proteins to be recruited, and finally 3) the downstream signaling pathways activated in the target cells. A1 and A3ARs inhibit the regulation of adenyl cyclase (AC) activity, while activation of the A2A and A2BAR subtypes stimulates AC, which leads to increases in cyclic AMP levels.^1^

ARs are widely distributed throughout the body and the fact that they are present in basically all cells mainly in the vessels, lungs, kidneys, heart, and parts of the brain makes them an interesting target for pharmacological intervention in many pathophysiological conditions linked to increased adenosine levels. There is evidence that A3ARs improve cellular antioxidant capacity, thus contributing to vasoprotection and reduction of cardiac myocyte death and strongly supporting an A3AR1-dependent cardioprotective response.^1^

In that number of the *Arquivos Brasileiros de Cardiologia* ^2^new N-acylhydrazone compounds that act on the adenosine system, compounds containing selenium, were evaluated. In particular, LASSBio-2062 was selected with agonist action, a potent vasodilator through the activation of A3 AR and K channels.

LASSBio-2062 (30 μmol/kg) reduced mean arterial pressure in SHR rats from 124.6 ± 8.6 to 72.0 ± 12.3 mmHg (p < 0.05). Activation of the subtype A3 adenosine receptor and potassium channels appears to be involved in the antihypertensive effect of LASSBio-2062. In conclusion, the new adenosine receptor agonist and potassium channel activator is a potential therapeutic agent for the treatment of systemic arterial hypertension.

Among the various biological functions promoted by selenium, antioxidant action has been described.^3^The activation of adenosine A3 receptors influences the activity of K+ channels, especially ATP-sensitive potassium channels, which can induce their opening,^4^which results in hyperpolarization and consequent blockade of Ca_2_+ channels. Reduced calcium influx leads to lower intracellular calcium concentration and results in vasodilation.^5^

The occurrence of bradycardia after intravenous administration of LASSBio-2062 may be beneficial due to the absence of the reflex tachycardia characteristics of many vasodilator medications which, in addition, cause volume retention requiring the association of beta-blockers and diuretics.

This study suggests that the adenosine system may be a new class of antihypertensive drugs in the arsenal of medications for the treatment of high blood pressure. One of the predicates for the benefit of antihypertensives cannot be attributed solely to the reduction in blood pressure. They must also have pleiotropic effects of endothelial and target organ protection present in LASSBio-2062^2^due to their antioxidant, anti-inflammatory, and atherosclerosis attenuation characteristics.

Most of the action of antihypertensives is associated with blockage or antagonism of hypertensive systems. Vasodilation attempts were made with angiotensin 1-7^6^agonists and possibly alamandine, compounds that oppose the vasoconstrictor angiotensin II. Adenosine receptor agonist action to promote vasodilation and reduce blood pressure in hypertensive humans is a challenge for LASSBio-2062 to consolidate itself as a new class of antihypertensives.
